# A data pipeline for secure extraction and sharing of social determinants of health

**DOI:** 10.1371/journal.pone.0317215

**Published:** 2025-01-31

**Authors:** Tyler Schappe, Lisa M. McElroy, Moronke Ogundolie, Roland Matsouaka, Ursula Rogers, Nrupen A. Bhavsar

**Affiliations:** 1 Department of Biostatistics and Bioinformatics, Duke University School of Medicine, Durham, North Carolina, United States of America; 2 Department of Population Health Sciences, Duke University School of Medicine, Durham, North Carolina, United States of America; 3 Department of Surgery, Duke University School of Medicine, Durham, North Carolina, United States of America; University of Lausanne, SWITZERLAND

## Abstract

**Objectives:**

Linking neighborhood- and patient-level data provides valuable information about the influence of upstream social determinants of health (SDOH). However, sharing of these data across health systems presents challenges. We set out to develop a pipeline to acquire, deidentify, and share neighborhood-level SDOH data across multiple health systems.

**Methods:**

We created a pipeline centered around Decentralized Geomarker Assessment for Multi-Site Studies (DeGAUSS) that utilizes containerization to geocode patient addresses and obtain neighborhood-level SDOH variables. We compared DeGAUSS to a third-party vendor geocoding tool available at Duke Health using a cohort of adult patients referred for abdominal transplant from January 1, 2016, to December 31, 2022. We calculated Cohen’s Kappa and percent disagreement at census block group and tract levels, and by Area Deprivation Index, urbanicity, and year.

**Results:**

The pipeline successfully generated SDOH data for 97.8% of addresses. There was high concordance between DeGAUSS and the vendor tool at the census block group (0.93) and tract levels (0.95). At the block group level, disagreement proportion differed by year and urbanicity, with larger disagreement in the rural category than in micropolitan and metropolitan categories (13%, 7%, 6.2%, respectively).

**Discussion and conclusion:**

We describe a novel pipeline that can facilitate the secure acquisition and sharing of neighborhood-level SDOH without sharing PHI. The pipeline can be scaled to include additional social, climate, and environmental variables, and can be extended to an unlimited number of health systems.

## Introduction

Social determinants of health (SDOH) are defined by the World Health Organization as “the conditions in which people are born, grow, live, work, and age, along with the wider set of forces and systems shaping the conditions of daily life” [1]. SDOH indicators are commonly assessed at the individual level, through measures like race, income and education level. However, neighborhood-level SDOH can provide additional important information about a patient’s social context, including living conditions, access to transportation, and community support. Assessment of SDOH indicators at multiple levels (e.g. patient, community, institution, etc.) provides an opportunity to examine upstream drivers of individual and population-level health inequities [2].

Incorporating neighborhood-level SDOH into research and quality improvement efforts presents unique challenges relative to other patient data. Within health systems, patient address data requires geocoding to link patients with public data sources that characterize neighborhood conditions, such as environmental quality, safety, or poverty [3]. Multicenter studies face an additional data security challenge because patient address is considered protected health information (PHI); obtaining the permissions and establishing the infrastructure for securely sharing PHI are costly and labor intensive.

Systematic approaches that securely link individual patients to neighborhood-level SDOH data can enable assessment of SDOH within health systems and the communities. It also allows examination of individual and community level SDOH across health systems, enabling the integration of community context into multi-center studies focused on care delivery and community context. Fundamental to these approaches is the need to accurately, reliably, and securely geocode patient addresses. Decentralized Geomarker Assessment for Multi-site Studies (DeGAUSS), is an open-source privacy-preserving software package that geocodes addresses in a standardized manner which can be used across multiple health systems. Yet, the DeGAUSS software suite includes limited area-level SDOH variables, and it lacks an automated pipeline to generate geocoded SDOH variables from address data. The goals of this study were to 1) develop a pipeline for generating community-level SDOH that augments the features provided by DeGAUSS and 2) compare DeGAUSS geocoding results to a vendor tool currently used by the Duke University Health System (DUHS) on a large, geographically diverse patient cohort in order to assess its viability for multi-health system analyses as a secure and standardized alternative to commercial geocoding software. Because geocoding accuracy directly affects downstream SDOH data and since previous work has found systematic differences in accuracy by rurality, we stratified our assessments by both rurality and by Area Deprivation Index (ADI); we also examined accuracy by year to elucidate potential temporal patterns [4].

## Materials and methods

### Data pipeline creation

**Fig 1:**
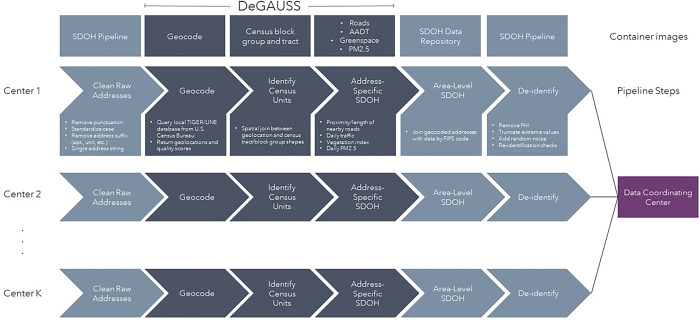
Conceptual diagram outlining the components of the generalized pipeline for geocoding patient addresses and obtaining de-identified social determinants of health (SDOH) data.

Our primary goal was to develop a pipeline for generating community-level SDOH that augments the features provided by DeGAUSS. We created a flexible in-house data pipeline centered around DeGAUSS v3.3.0 that utilizes containerization via Singularity [5] to geocode patient addresses and obtain neighborhood-level SDOH variables. The pipeline requires a comma-separate values (CSV) file of address text as input and provides a CSV file with each address and associated de-identified SDOH variables as output. The pipeline checks for the existence of the required Singularity containers and pulls them from the Duke public container registry if needed. After container verification, the pipeline performs the following steps, checking for successful output after each: 1) normalize input address formatting, 2) geocode address using the 2021 TIGER/Line shapefiles via DeGAUSS, 3) identify census tract and block group level FIPS codes, 4) merge with a curated SDOH data repository, and 5) de-identify output data (Fig 1). The address normalization step standardizes formatting beyond street numbers, such as apartment units or suites. The SDOH data repository is a publicly-available auxiliary resource [6] created by compiling various publicly-available SDOH data sources by spatial scale; at present it contains 77 distinct fields and it can be expanded depending on the research question in future studies. Additional de-identification steps can also be added, including removal of PHI, outlier truncation, and the addition of random noise to continuous variables.

### Data sources

#### Electronic health records

We obtained address data from the Duke University Health System (DUHS) for a cohort of adult patients referred for abdominal transplant between January 1, 2016 and December 31, 2022. DUHS is a tertiary health system that consists of three hospitals and a network of primary and specialty care clinics. This study was approved by the Duke University IRB; data were accessed on March 1, 2023 and the authors had access to information that could identify individual participants during and after data collection, with IRB permission. The need for consent was waived by the DUHS IRB.

#### Area deprivation index

Neighborhood socioeconomic status was quantified with the Area Deprivation Index (ADI). The ADI is a composite score that ranks neighborhoods by socioeconomic disadvantage, based on factors related to employment, income, and education at state and national levels [7]. We assigned quintile categories to each geocoded address based on the 2018 national percentile ranking. Addresses in census units with no ADI value were assigned to a separate ‘missing’ category.

#### Rural-urban commuting area

We classified each address into one of five urban/rural categories based on the 2010 Rural-Urban Commuting Area (RUCA) classifications. The 2010 RUCA codes published by the USDA Economic Research Services (ERS) classify census tracts using measures of population density, urbanization, and daily commuting.

### Geocoding methods

#### Duke university health system (DUHS) geocoding

DUHS uses a third-party vendor geocoding tool to provide census unit assignments from patient addresses. The tool is used for health system-level geocoding of current and historic addresses for DUHS patients.

#### DeGAUSS geocoding

DeGAUSS is an open-source software suite that was developed specifically for facilitating geocoding of patient addresses for multi-site studies. It provides secure geocoding functionality that uses a local instance of the TIGER/Line Shapefiles database provided by the US Census Bureau [8]. DeGAUSS assigns census tract and block group level FIPS codes based on address geolocations. The security of DeGAUSS is based on containerization, a system-level virtualization of a pre-built compute environment that includes software tools and databases. Containerization allows workflows to be executed within an isolated compute instance using self-contained tools and databases, preventing exposure of PHI outside of the compute environment. The compute environment is encapsulated in a single file (‘container’) which can easily be run on a variety of computational infrastructures, including secure enclaves designed for handling PHI. In the case of DeGAUSS, the required software along with the U.S. Census TIGER/Line Shapefiles database are built into the container, precluding the need to query remote databases and share PHI to obtain geolocation based SDOH.

### Statistical analysis

Our secondary goal was to examine overall agreement between geocoding output from DeGAUSS and the third-party vendor geocoding tool. To accomplish this, we first quantified concordance with respect to census unit assignment (i.e. the census tract and block group Federal Information Processing Standards (FIPS) code assignment based on the address geolocation). We hypothesized that concordance may differ by neighborhood socioeconomic status, by urban-rural category, and by time. A second way in which we characterized agreement was the geographic distance between the geocoded locations provided by each tool. Prior to analysis, we processed the raw geocoding output for the patient cohort address data using DeGAUSS and the vendor tool. We combined the raw DeGAUSS geocoding results with the original address dataset that contained the vendor tool geocoding results. We excluded addresses that were not successfully geocoded by both tools and collapsed duplicative addresses into the first instance the address was reported.

#### Overall concordance

We compared the performance of DeGAUSS to the vendor tool by quantifying the concordance of census unit assignments at both the census tract and census block group levels. To do so, we utilized two metrics: Cohen’s kappa [9], a commonly-used index that accounts for agreement that is expected to occur by random chance, and percent disagreement. Because of the large number of categories for classification, we calculated Cohen’s kappa following the generalized equations outlined in Pontius and Millones, 2011 [10]. To examine proportion disagreement, we first derived a binary outcome that indicates whether the census unit assignments differed among the tools. We then fit an intercept-only logistic regression model using this binary outcome to estimate percent disagreement (as the estimated probability) and corresponding 95% confidence intervals for both census tract and block group levels.

#### Heterogeneity of concordance by strata

In addition to overall concordance, we quantified differences in concordance of census unit assignments among strata for urban/rural categories, socioeconomic deprivation categories, and the first calendar year that the address was valid for the patient. We calculated the corresponding Cohen’s Kappa by strata and used a Bonferroni correction [11] to account for multiple comparisons in the resulting p-values [12]. We then examined whether percent disagreement differed by strata by fitting a logistic regression model using the same outcome as above except with one stratification variable as a covariate. We used a likelihood ratio test to compare this full model to the previously fit intercept-only model to assess the evidence for differences in percent disagreement among strata. We also used the full model to estimate percent disagreement and corresponding Bonferroni-adjusted 95% confidence intervals for each stratum. In cases with a significant likelihood ratio test (i.e., p < 0.05), we performed follow-up t-tests for all pairwise comparisons among all strata and used Tukey adjustment for the resulting p-values [13].

#### Geographic distance by strata

Because distance to transplant center is a commonly studied SDOH, we used geographic distance between geocoded locations provided by each tool for each address as a supplementary metric to compare their performance [14–17]. We first calculated the straight-line geographic distances between geolocations provided by each tool for all addresses [18]. We then calculated median distances and performed non-parametric Kruskal-Wallis tests for differences in the distribution of distances by strata. In the case of a significant Kruskal-Wallis test, we performed Wilcoxon rank sum tests for all pairwise comparisons among strata and used the Bonferroni adjustment method to account for multiple comparisons in the resulting p-values and 95% confidence intervals. Data analysis was performed using the R project for statistical computing v4.3.1 [19].

## Results

A total of 13,562 unique addresses associated with patients referred for an abdominal transplant at DUHS from January 1, 2016, to December 31, 2022, were geocoded by the third-party vendor geocoding tool, of which 13,262 (97.8%) DeGAUSS was able to geocode successfully. The population of addresses originated from 42 different U.S. states, with 10,209 (75.3%) being from metropolitan areas, 1,991 (14.7%) from micropolitan locations, 845 (6.2%) from small towns, 239 (1.8%) from rural regions, and 278 (2.0%) missing a RUCA designation. With respect to ADI, 974 (7.2%) addresses were in the least deprived quintile of block groups nationally, 2,328 (17.2%) were in the 2^nd^ quintile, 3,294 (24.3%) were in the 3^rd^ quintile, 3,567 (26.3%) were in the 4^th^ quintile, 3,222 (23.8%) were in the most highly deprived quintile of block groups, and 177 (1.3%) of addresses had an undefined ADI.

Overall, at census block group and census tract levels, there was high agreement as measured by Cohen’s kappa (0.93 and 0.95, respectively) and low percent disagreement (6.7% and 4.6%, respectively) between the vendor tool and DeGAUSS in census unit assignments (Table 1).

**Table 1:** Estimated Cohen’s Kappa and percent disagreement [95% confidence interval] of census tract and block group Federal Information Processing Standards (FIPS) assignments resulting from DeGAUSS and vendor tool geocoding process, by geographic census unit.

Geography	Est. Cohen’s Kappa [95% CI]	Est. % disagreement [95% CI]
Block group	0.93 [0.93, 0.94]	6.7 [6.2, 7.1]
Tract	0.95 [0.95, 0.96]	4.6 [4.3, 5]

The overall percent disagreement between DeGAUSS and the vendor tool differed significantly by urbanicity at the block group level (*P* < 0.001) but not at the census tract level (*P* > 0.98, Table 2).

**Table 2:** Results of likelihood ratio tests for differences in percent disagreement among strata of census tract and block group assignments between DeGAUSS and vendor tool geocoding.

Geography	Stratification variable	Deviance	p-value
Block group	Urban/rural category	23.4	< 0.001
	Calendar year address was valid	26.5	0.002
	ADI quintile	8.5	0.52
Tract	Urban/rural category	9.4	0.10
	Calendar year address was valid	16.4	0.09
	ADI quintile	6.6	> 0.99

*P*-values were adjusted for multiple stratification variables within geography using the Bonferroni method.

The percent disagreement differed by the calendar year that the address was applicable to the patient at the block group level (*P* < 0.003) but not at the tract level (*P* > 0.08, Table 2). There was no heterogeneity in percent disagreement by ADI quintiles at either block group (*P* > 0.52) or tract (*P* > 0.99, Table 2) levels.

### Concordance by Urban-rural

In analyses stratified by urbanicity, there was greater estimated concordance in geocoding in urban areas as compared to rural areas (Table 3).

**Table 3:** Estimated Cohen’s Kappa and percent disagreement of census tract and block group Federal Information Processing Standards (FIPS) assignments resulting from DeGAUSS and vendor tool geocoding process, stratified by urban/rural category.

Geography	Urban/rural category	Num. addresses	Est. Cohen’s Kappa [95% CI*]	Est. % disagreement [95% CI*]
Block group	Metropolitan	10,192	0.94 [0.93, 0.94]	6.2 [5.6, 6.8]
	Micropolitan	1,988	0.93 [0.92, 0.94]	7.0 [5.7, 8.6]
	Small town	843	0.91 [0.88, 0.93]	9.1 [6.9, 11.9]
	Rural	239	0.87 [0.81, 0.92]	13.0 [8.4, 19.4]
Tract	Metropolitan	10,192	0.96 [0.95, 0.96]	4.3 [3.9, 4.9]
	Micropolitan	1,988	0.95 [0.94, 0.96]	5.1 [4, 6.5]
	Small town	843	0.94 [0.92, 0.96]	6.0 [4.3, 8.5]
	Rural	239	0.93 [0.88, 0.97]	7.1 [3.9, 12.6]

*Bonferroni-adjusted confidence intervals among strata within each census unit geography.

**Fig 2:**
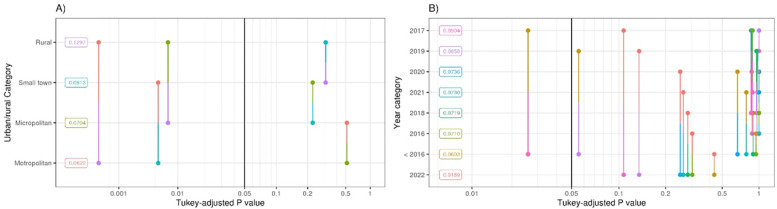
Pairwise comparisons of probability of disagreement of census unit assignments between DeGAUSS and vendor tool geocoding at the block group level among A) urban/rural categories and B) calendar year address was applicable.

Metropolitan areas had a high estimated index of agreement (0.94; 95% CI: [0.93, 0.94]) and a low estimated percent disagreement (6.2%; 95% CI: [5.6%, 6.8%]) at the block group and census tract level (index of agreement: 0.96, 95% CI: [0.95, 0.96]; percent disagreement: 4.3%, 95% CI: [3.9%, 4.9%]). By contrast, rural addresses had a lower estimated index of agreement at the block group and tract levels (0.87 and 0.93, respectively) and a higher estimated percent disagreement (13.0% and 7.1%, respectively), the latter of which was a statistically significant pairwise contrast (*P* < 0.001, Fig 2A). Moreover, estimated percent disagreement among rural addresses was significantly higher (P < 0.008) than among micropolitan addresses (7.0%, 95% CI: [5.7%, 8.6%]) at the census block group level. Additionally, estimated percent disagreement among ‘small town’ addresses (9.1%, 95% CI: [6.9%, 11.9%]) was significantly higher (*P* < 0.006) than for metropolitan addresses (6.2%, 95% CI: [5.6%, 6.8%], Fig 2A).

### Concordance by address year

In analyses stratified by the first calendar year that the address was observed, estimated percent disagreement between the geocoders at the block group level was significantly different between addresses first observed prior to 2016 (6.0% disagreement (95% CI: [5.3%, 6.8%])) as compared to addresses first observed in 2017 (9.0% disagreement (95% CI: [6.6%, 12.3%], *P* < 0.026, Fig 2B). There was no evidence for heterogeneity in percent disagreement by year at the tract level (*P* > 0.05, Table 2).

### Geographic distance

For most addresses (92%), the distance between geocoded locations provided by each tool was less than 1,000 ft (S1 Table). However, 4% of addresses had distances greater than 1 mile, including 0.95% of metropolitan addresses with distances greater than 10 miles (S1 Fig, S1 Table). The distributions of geographic distances between the geocoding tools were right skewed, as well, within urban-rural categories (S1 Table) and within ADI quintile categories (S2 Table). There were significant differences in the distances between geocoded locations provided by the vendor tool and DeGAUSS by urban/rural categories (*P* < 0.001, S3 Table). There were significant shifts (i.e. stochastic dominance) among the distributions of geographic distances for all pairwise comparisons among urban/rural categories except for the ‘small town’ vs ‘rural’ comparison (S4 Table). For example, the estimated median difference in geographic distance for rural addresses compared to metropolitan addresses was 93.3 ft. (95% CI: 63.6 ft., 127.7 ft.).

There were significant differences in the distances between geocoded locations provided by the vendor tool and DeGAUSS by ADI quintile (*P* < 0.014, S2 Table), with the largest median distance in the 4th ADI quintile of 140 ft (S2 Table). Among all pairwise comparisons for stochastic dominance, the only statistically significant result was between the 2^nd^ and 4^th^ quintile groups (*P* < 0.01), but this is unlikely to be practically meaningful as the estimated median difference in distance between the two quintiles was 8.79 ft. (95% CI: [3.62 ft., 14.52 ft.]) (S5 Table).

## Discussion

Neighborhood-level SDOH are increasingly recognized as upstream drivers of health disparities [20–22], but can be challenging to include in analyses of EHR data because of privacy concerns. For projects that include multiple health systems, generating and sharing neighborhood SDOH requires either expertise with geocoding at each site and ensuring geocoding practices are the same across sites, or designating one site as a coordinating center, obtaining IRB approval and creating data use agreements that allow sharing of PHI. There is a need to develop reproducible privacy-preserving methods that allow for incorporation of community level SDOH into multicenter research without the need to share PHI [23–26].

We created a generalized data pipeline using DeGAUSS to provide consistent geocoding and generation of neighborhood based SDOH data for sharing across health systems without sharing PHI. These features can facilitate multi-health system participation of large-scale policy relevant research, such as the impact of climate on health or state subsidies for areas lacking referring providers, by allowing the inclusion of geographically diverse populations. The pipeline can be shared with an unlimited number of health systems and its flexibility enables future extensions to be introduced. Additional SDOH variables can be included and easily pushed to participating centers, including raster data such as Normalized Difference Vegetation Index (NDVI); the complexity of this process depends on the variable source and type. Further, customized definitions of neighborhoods can be utilized assuming corresponding polygons exist. We note that ensuring correct temporality of exposures relative to health outcomes is not ensured by DeGAUSS or our pipeline, but rather falls to the investigator or analyst.

We assessed the accuracy of DeGAUSS geocoding by comparing the resulting census tract and block group assignments to those provided by the third-party vendor geocoding tool available at DUHS. DeGAUSS was able to geocode and provide census assignments for 97.8% and demonstrated higher agreement with a third-party vendor geocoding tool at the census tract level compared to the block group level. There was no heterogeneity in percent disagreement among ADI quintiles at either tract or block group levels, indicating that DeGAUSS can be used effectively across socioeconomic strata. However, we found a higher level of disagreement for rural addresses compared with both micropolitan and metropolitan categories, and “small-town” addresses as compared to metropolitan addresses. This was supported by geographic distances between geocoded locations, which were larger for rural and small-town addresses compared to suburban and urban areas. This is not unexpected given differences in geographic scales and mail delivery infrastructure such as rural route numbers, and consistent with prior research demonstrating a relationship between rurality and geocoding ability [27]. Higher disagreement in geocoding tools may pose a challenge when examining urban/rural disparities. There is a tradeoff between greater address inclusion, which can decrease selection bias, and decreased geocoding accuracy, which increases information bias. Future work should examine more advanced methods of characterizing social context within rural geographic regions.

We observed moderate discordance between DeGAUSS and the third-party vendor geocoding tool in urban and suburban areas; surprisingly, nearly 100 metropolitan addresses had distances of greater than 10 miles between the geocoded locations. This may be indicative of geocoding failure for at least one of the tools, but without a validated gold standard to compare against, it is not possible to determine which tool is correct. One potential explanation could be an anti-conservative string search algorithm used to query the input addresses given the absence of a perfect match. For example, it’s possible that one tool is matching addresses not included in its database to an incorrect address in a different city or town that shares common features with the query, such as a street name. While this geocoding approach has the potential to result in large error rates, only a small fraction of addresses in our cohort were affected.

There are some limitations to note. First, there is no definitive way to determine which geocoding tool (or neither) provides the true correct geocoded location. We hypothesize that short distances between geocoded addresses is small (e.g., 50 feet), indicate high accuracy in geocoding [12,28]. Second, there are several products on the market to facilitate geocoding of patient address data. We compared DeGAUSS to the DUHS third-party geocoding tool with good results, but performance may vary across other products and software.

## Conclusion

Incorporating neighborhood-level SDOH into research and quality improvement efforts can identify upstream drivers of disparities in care ripe for intervention. Unfortunately, SDOH data are housed across multiple national data sources and organized at varying geographic levels. We describe a novel pipeline that is driven by DeGAUSS, an open-source software application capable of geocoding patient addresses and SDOH data without the need to share PHI. We found that DeGAUSS can address these concerns and is an accurate, open source, privacy-preserving tool that can facilitate incorporation of community level SDOH into multicenter studies that use EHR data.

## Supporting information

S1 Fig Number of addresses by distance (mi.) between geocoded locations stratified by urban-rural category for addresses with distances of greater than 2 miles. (DOCX)

S1 Table Percentage of addresses by geographic distance between geocoded locations provided by DeGAUSS and the vendor tool geocoder, stratified by urban-rural category. (DOCX)

S2 Table Distance (ft.) between geocoded locations provided by DeGAUSS and vendor tool, stratified by quintiles of Area Deprivation Index. (DOCX)

S3 Table Distance (ft.) between geocoded locations provided by DeGAUSS and vendor tool, stratified by urban-rural category. (DOCX)

S4 Table Estimated median difference in distance between samples of addresses drawn from each group in the comparison by urban-rural category. (DOCX)

S5 Table Estimated median differences in distance between samples of addresses drawn from each group in the comparison by quintile of Area Deprivation Index (ADI). (DOCX)

## Abbreviations

ADI: Area Deprivation Index
DeGAUSS: Decentralized Geomarker Assessment for Multi-site Studies
DUHS: Duke University Health System
ERS: Economic Research Service
FIPS: Federal Information Processing Standards
PHI: Protected health information
RUCA: Rural-Urban Commuter Area
SDOH: Social determinants of health
TIGER: Topologically Integrated Geographic Encoding and Referencing
USDA: United States Department of Agriculture
