# Supplementary material for: A data pipeline for secure extraction and sharing of social determinants of health
Source: PLoS One. 2025 Jan 31;20(1):e0317215. doi: 10.1371/journal.pone.0317215 (PMC11785280; doi:10.1371/journal.pone.0317215)

**Figure S1.** Number of addresses by distance (mi.) between geocoded locations stratified by urban-rural category for addresses with distances of greater than 2 miles.
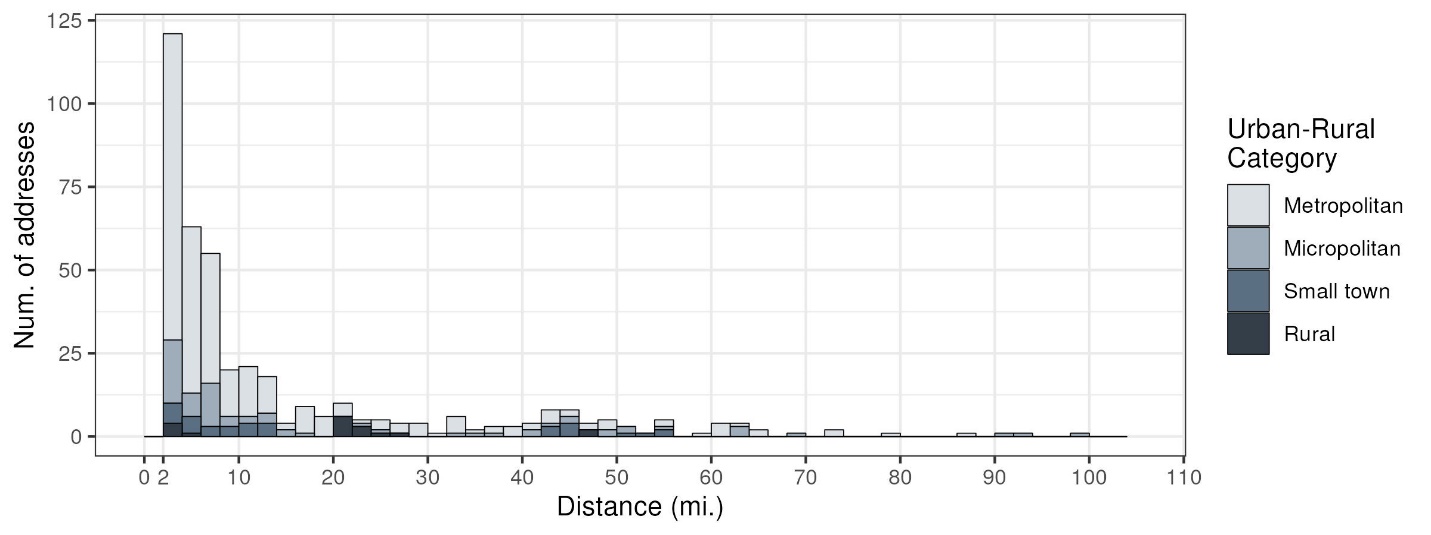

Supplement: S1 Fig — (DOCX) [file pone.0317215.s001.docx]
