# Supplementary material for: A data pipeline for secure extraction and sharing of social determinants of health
Source: PLoS One. 2025 Jan 31;20(1):e0317215. doi: 10.1371/journal.pone.0317215 (PMC11785280; doi:10.1371/journal.pone.0317215)
Supplement: S1 Table — (DOCX) [file pone.0317215.s002.docx]

**Table S1.** Percentage of addresses by geographic distance between geocoded locations provided by DeGAUSS and the vendor tool geocoder, stratified by urban-rural category.

| **Distance** | **Percent of Addresses** | | | | |
| --- | --- | --- | --- | --- | --- |
|  | **Metropolitan** | **Micropolitan** | **Small town** | **Rural** | **Overall** |
| 0 - 50 ft. | 32.25 | 27.72 | 22.3 | 14.64 | 30.62 |
| > 50 ft. - 100 ft. | 13.27 | 14.59 | 12.34 | 12.13 | 13.38 |
| > 100 ft. - 250 ft. | 22.17 | 23.69 | 20.17 | 23.43 | 22.3 |
| > 250 ft. - 500 ft. | 17.11 | 15.44 | 18.03 | 15.48 | 16.89 |
| > 500 ft. - 1,000 ft. | 8.38 | 9.05 | 11.03 | 15.48 | 8.78 |
| > 1,000 ft. - 0.5 mi. | 2.49 | 4.12 | 7.47 | 6.28 | 3.12 |
| > 0.5 mi. - 1 mi. | 0.72 | 1.21 | 3.08 | 1.26 | 0.95 |
| > 1 mi. - 5 mi. | 1.92 | 1.81 | 2.49 | 5.44 | 2.01 |
| > 5 mi. - 10 mi. | 0.74 | 0.86 | 0.83 | 0.42 | 0.75 |
| > 10 mi. - 100 mi. | 0.94 | 1.51 | 2.25 | 5.44 | 1.19 |
| > 100 mi. | 0.01 | 0 | 0 | 0 | 0.01 |
