# Supplementary material for: A data pipeline for secure extraction and sharing of social determinants of health
Source: PLoS One. 2025 Jan 31;20(1):e0317215. doi: 10.1371/journal.pone.0317215 (PMC11785280; doi:10.1371/journal.pone.0317215)
Supplement: S2 Table — (DOCX) [file pone.0317215.s003.docx]

**Table S2.** Distance (ft.) between geocoded locations provided by DeGAUSS and vendor tool, stratified by quintiles of Area Deprivation Index

|  | **1st (N=952)** | **2nd (N=2271)** | **3rd (N=3231)** | **4th (N=3508)** | **5th (N=3180)** | **Missing (N=120)** | **p-value** |
| --- | --- | --- | --- | --- | --- | --- | --- |
| **Distance Between Geocoded Locations (ft.)** |  |  |  |  |  |  |  |
| Mean  (SD) | 2610 (22043) | 2382 (17017) | 2532 (18558) | 2935 (24449) | 3814  (32913) | 2594 (24063) | 0.0135 |
| Median  (Min, Max) | 124  (0, 320096) | 127  (0, 276024) | 136  (0, 456945) | 140  (0, 527678) | 132  (0, 1174018) | 116  (0, 263746) |  |

p-value is from Kruskal-Wallis test for stochastic dominance in at least one ADI quintile.
