# Supplementary material for: A data pipeline for secure extraction and sharing of social determinants of health
Source: PLoS One. 2025 Jan 31;20(1):e0317215. doi: 10.1371/journal.pone.0317215 (PMC11785280; doi:10.1371/journal.pone.0317215)
Supplement: S3 Table — (DOCX) [file pone.0317215.s004.docx]

**Table S3.** Distance (ft.) between geocoded locations provided by DeGAUSS and vendor tool, stratified by urban-rural category

|  | **Metropolitan (N=10192)** | **Micropolitan (N=1988)** | **Small town (N=843)** | **Rural (N=239)** | **p-value** |
| --- | --- | --- | --- | --- | --- |
| **Distance Between Geocoded Locations (ft.)** |  |  |  |  |  |
| Mean (SD) | 2374 (21770) | 4227 (31771) | 4913 (29329) | 8653 (33488) | <0.001 |
| Median  (Min, Max) | 126  (0, 1174018) | 141  (0, 527678) | 199  (1, 295159) | 249  (5, 247889) |  |

p-value is from Kruskal-Wallis test for stochastic dominance in at least one urban-rural category.
