# Supplementary material for: A data pipeline for secure extraction and sharing of social determinants of health
Source: PLoS One. 2025 Jan 31;20(1):e0317215. doi: 10.1371/journal.pone.0317215 (PMC11785280; doi:10.1371/journal.pone.0317215)
Supplement: S4 Table — (DOCX) [file pone.0317215.s005.docx]

**Table S4.** Estimated median difference in distance between samples of addresses drawn from each group in the comparison by urban-rural category

| **Urban-Rural Comparison** | **Distance (Feet) [95% CI]** | **p-value*** |
| --- | --- | --- |
| Micropolitan - Metropolitan | 9.77 [4.96, 14.79] | < 0.001 |
| Rural - Metropolitan | 93.29 [63.6, 127.66] | < 0.001 |
| Rural - Micropolitan | 78.41 [47.09, 113.21] | < 0.001 |
| Small town - Metropolitan | 45.02 [32.33, 60.52] | < 0.001 |
| Small town - Micropolitan | 31.89 [18.99, 48.01] | < 0.001 |
| Small town - Rural | 34.89 [5.79, 71.83] | 0.099 |

*Bonferroni corrected p-value
