# Supplementary material for: A data pipeline for secure extraction and sharing of social determinants of health
Source: PLoS One. 2025 Jan 31;20(1):e0317215. doi: 10.1371/journal.pone.0317215 (PMC11785280; doi:10.1371/journal.pone.0317215)
Supplement: S5 Table — (DOCX) [file pone.0317215.s006.docx]

**Table S5.** Estimated median differences in distance between samples of addresses drawn from each group in the comparison by quintile of Area Deprivation Index (ADI)

| **Pairwise ADI Quintile Comparison** | **Distance (Feet) [95% CI]** | **p-value*** |
| --- | --- | --- |
| 2nd - 1st | 0.45 [-5.97, 7.22] | 1.00 |
| 3rd - 1st | -2.9 [-9.69, 3.56] | 1.00 |
| 3rd - 2nd | -3.34 [-8.22, 1.23] | 1.00 |
| 4th - 1st | -8.15 [-16.14, -1.21] | 0.31 |
| 4th - 2nd | -8.79 [-14.52, -3.62] | 0.01 |
| 4th - 3rd | -5.18 [-10.22, -0.56] | 0.41 |
| 5th - 1st | -3.6 [-10.6, 3.26] | 1.00 |
| 5th - 2nd | -4.23 [-9.29, 0.63] | 1.00 |
| 5th - 3rd | -0.77 [-5.41, 3.89] | 1.00 |
| 5th - 4th | 4.55 [-0.3, 9.68] | 1.00 |
| Missing - 1st | 1.95 [-16.13, 23.99] | 1.00 |
| Missing - 2nd | 1.44 [-15.69, 20.89] | 1.00 |
| Missing - 3rd | 5.37 [-11.93, 25.93] | 1.00 |
| Missing - 4th | 11.69 [-6.97, 35.27] | 1.00 |
| Missing - 5th | 5.895 [-12.05, 26.63] | 1.00 |

*Bonferroni adjusted p-value
